# Carotid corrected flow time as a sensitive indicator for early diagnosis and dynamic monitoring of hemorrhagic shock: A translational study from porcine model to clinical practice

**DOI:** 10.1016/j.cjtee.2026.04.003

**Published:** 2026-05-20

**Authors:** Huan Yang, Hao Tang, Yao Xiao, Yingying Zhang, Xiao Chen, Chang Liu, Yi Zhang, Yusheng Zhang, Jingqin Fang, Lianyang Zhang, Tao Li, Dong Han

**Affiliations:** a Department of Ultrasound Diagnosis, Daping Hospital, Army Medical University, Chongqing, 400042, China; b Department of Critical Care Medicine, Daping Hospital, Army Medical University, Chongqing, 400042, China; c Department of Traumatology, Daping Hospital, Army Military Medical University, Chongqing, 400042, China; d Department of Medical Imaging Teaching and Research, Daping Hospital, Chongqing, 400042, China

**Keywords:** Hemorrhagic shock, Carotid corrected flow time (CCFt), Carotid ultrasound, Hemodynamic monitoring, Volume status

## Abstract

**Purpose:**

Early identification of hemorrhagic shock remains a clinical challenge, as traditional vital signs often fail to detect hypovolemia during the compensatory phase. Carotid corrected flow time (CCFt), derived from Doppler ultrasound, has been proposed as a surrogate for stroke volume. However, its precise correlation with quantitative blood loss and its diagnostic value in the early stages of shock remain to be fully elucidated. This study aimed to systematically evaluate the sensitivity and diagnostic efficacy of CCFt in graded hemorrhagic shock through both animal experiments and clinical validation.

**Methods:**

The study consisted of 2 phases. In the animal phase, a controlled, graded hemorrhage model was established in 7 Bama miniature pigs. Hemodynamic parameters and carotid ultrasound indices (CCFt, peak systolic velocity, and diameter) were measured at baseline and after cumulative blood loss intervals (0 – 1000 mL). In the clinical phase, a case-control study was conducted comparing 18 patients (admitted between November 2024 and February 2026) with hemorrhagic shock to 36 non-shock controls. Diagnostic accuracy was assessed using receiver operating characteristic curve analysis, and dynamic changes in CCFt were monitored during fluid resuscitation in representative cases.

**Results:**

In the animal model, CCFt demonstrated a strong negative linear correlation with quantitative blood loss (*R* = 0.870, *p* < 0.001). Notably, CCFt decreased significantly at the early stage of hemorrhage (<300 mL), exhibiting high diagnostic accuracy for early/mild shock (area under the curve (AUC) = 0.900). In the clinical cohort, CCFt was significantly lower in the shock group than in controls (*p* < 0.001), yielding an AUC of 0.991 for the diagnosis of hemorrhagic shock, with an optimal cutoff of 311 ms. Longitudinal monitoring revealed that changes in CCFt were highly concordant with clinical response to fluid resuscitation and lactate clearance.

**Conclusion:**

CCFt is a sensitive, non-invasive indicator of blood volume loss that correlates closely with hemorrhage severity and enables early detection of compensated hemorrhagic shock. CCFt may serve as a valuable adjunctive tool for bedside assessment and precision-guided resuscitation in emergency and critical care settings.

## 1 Introduction

Hemorrhagic shock remains a leading cause of mortality in trauma patients worldwide, accounting for approximately 30% to 40% of trauma-related deaths.^1^^,^^2^ Successful treatment depends on timely recognition of hypovolemia within the “golden hour” and precise fluid resuscitation to restore tissue perfusion. However, during the early compensatory phase of shock, neurohumoral regulation mechanisms maintain blood pressure (BP) and heart rate (HR) within relatively normal ranges, effectively masking underlying tissue hypoperfusion and challenging the accurate clinical assessment of effective circulating volume.^3^ Conventional non-invasive vital signs (e.g., BP, HR) typically do not exhibit significant abnormalities until blood loss exceeds 30% of the total blood volume (decompensated phase). This diagnostic lag can lead to delayed resuscitation, thereby increasing the risk of adverse outcomes.^4^^,^^5^ Therefore, there is an urgent need for an early, sensitive, and bedside-applicable hemodynamic indicator to guide early resuscitation decisions in hemorrhagic shock.

Point-of-care ultrasound has become a vital tool for assessing hemorrhagic shock. While inferior vena cava diameter and its respiratory variation are widely used to assess fluid responsiveness, their accuracy is often compromised by factors such as obesity, intra-abdominal hypertension, intestinal gas, or abdominal trauma.^1^^,^^5^^,^^6^ In contrast, the carotid artery offers a distinct advantage due to its superficial location, allowing for the easy acquisition of stable images with low subjective bias. It is increasingly recognized as a novel acoustic window for shock assessment in emergency or combat settings.^5^ Corrected carotid flow time (CCFt), a parameter derived from Doppler ultrasound representing the HR-corrected left ventricular ejection time, has been suggested as a surrogate for stroke volume. Previous studies indicate that CCFt reflects volume status well and holds potential value in predicting fluid responsiveness.^5^^,^^7^, ^8^, ^9^, ^10^

Despite this, systematic data on the precise correlation between CCFt and quantitative blood loss, particularly the dynamic patterns of evolution during graded hemorrhage, remain lacking. Furthermore, whether the diagnostic efficacy of CCFt in the early (compensatory) stage of hemorrhagic shock is superior to traditional vital signs has not been fully validated in clinical practice. Consequently, this study aimed to systematically evaluate the clinical utility of CCFt through both experimental and clinical approaches: first, by establishing a precise graded hemorrhagic shock model in swine to characterize CCFt changes across different hemorrhage stages (especially early stages) and quantify its correlation with blood loss; and second, by validating the diagnostic performance of CCFt in distinguishing hemorrhagic shock in a real-world emergency clinical setting via a case-control study. We hypothesized that CCFt is more sensitive to changes in blood volume than traditional indicators, providing an objective basis for the early identification and “precision resuscitation” of hemorrhagic shock.

## 2 Methods

### 2.1 Animal experiment

#### 2.1.1 Experimental animals

Seven healthy male Bama miniature pigs (weight: (29.5 ± 3.1) kg) were provided by the Laboratory Animal Center of the Army Medical Center. The animal research protocol was approved by the Animal Welfare and Ethical Review Committee of the Army Medical University (Approval No. 20257051). All animal experiments were conducted in accordance with the National Institutes of Health Guide for the Care and Use of Laboratory Animals. Inclusion criteria: good general health with no underlying diseases; Exclusion criteria: pre-existing coagulation disorders or hemorrhagic diseases.

#### 2.1.2 Model construction

**Fig. 1:**
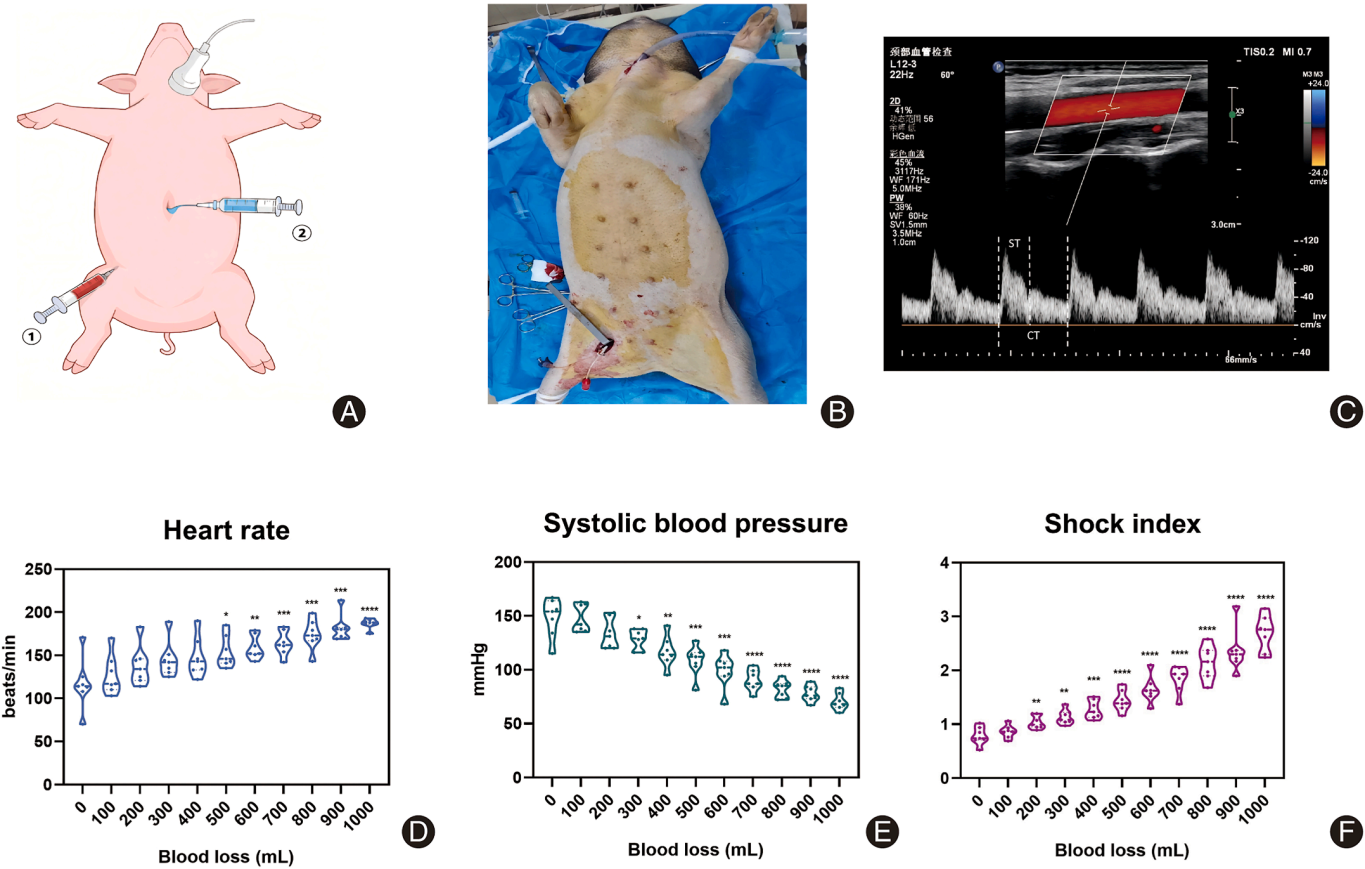
Construction of the experimental animal model and methods for carotid ultrasound measurement, as well as hemodynamic validation of the hemorrhagic shock model. (A) Schematic diagram of catheterization in Bama miniature pigs: ① Indicates femoral artery catheterization, utilized for graded blood withdrawal to establish the hemorrhage model; ② Indicates intraperitoneal catheterization, utilized for the reinjection of autologous blood into the abdominal cavity to simulate hemoperitoneum and the pathophysiology of hemorrhagic shock. (B) Illustration of the hemorrhagic shock model induced by intraperitoneal bleeding. (C) Schematic representation of carotid hemodynamic parameter measurement. The upper panel shows the long-axis 2-dimensional ultrasound image of the CCA, with the sampling volume placed in the center of the vascular lumen and the angle between the ultrasound beam and blood flow adjusted to <60°; the lower panel displays the corresponding pulsed-wave Doppler spectrum. Markers illustrate the measurement of time intervals: ST, measured from the onset of the systolic upstroke to the dicrotic notch; CT, representing one cardiac cycle. CCFt was calculated using Bazett's formula: CCFt = ST/ $\sqrt{\mathrm{CT}}$. ST was measured in milliseconds (ms), and CT was measured in seconds (s). (D-F) Line graph illustrating the trends of heart rate, systolic blood pressure, and shock index relative to cumulative blood loss (0 – 1000 mL). The graphs show a progressive upward trend in HR and SI and a declining trend in SBP as blood loss increases. Statistical significance versus baseline (0 mL): ∗*p* < 0.05, ∗∗*p* < 0.01, ∗∗∗*p* < 0.001, ∗∗∗∗*p* < 0.0001. CCA: common carotid artery; ST: systolic time; CT: cycle time; CCFt: carotid corrected flow time; HR: heart rate; SI: shock index; SBP: systolic blood pressure.

**Fig. 2:**
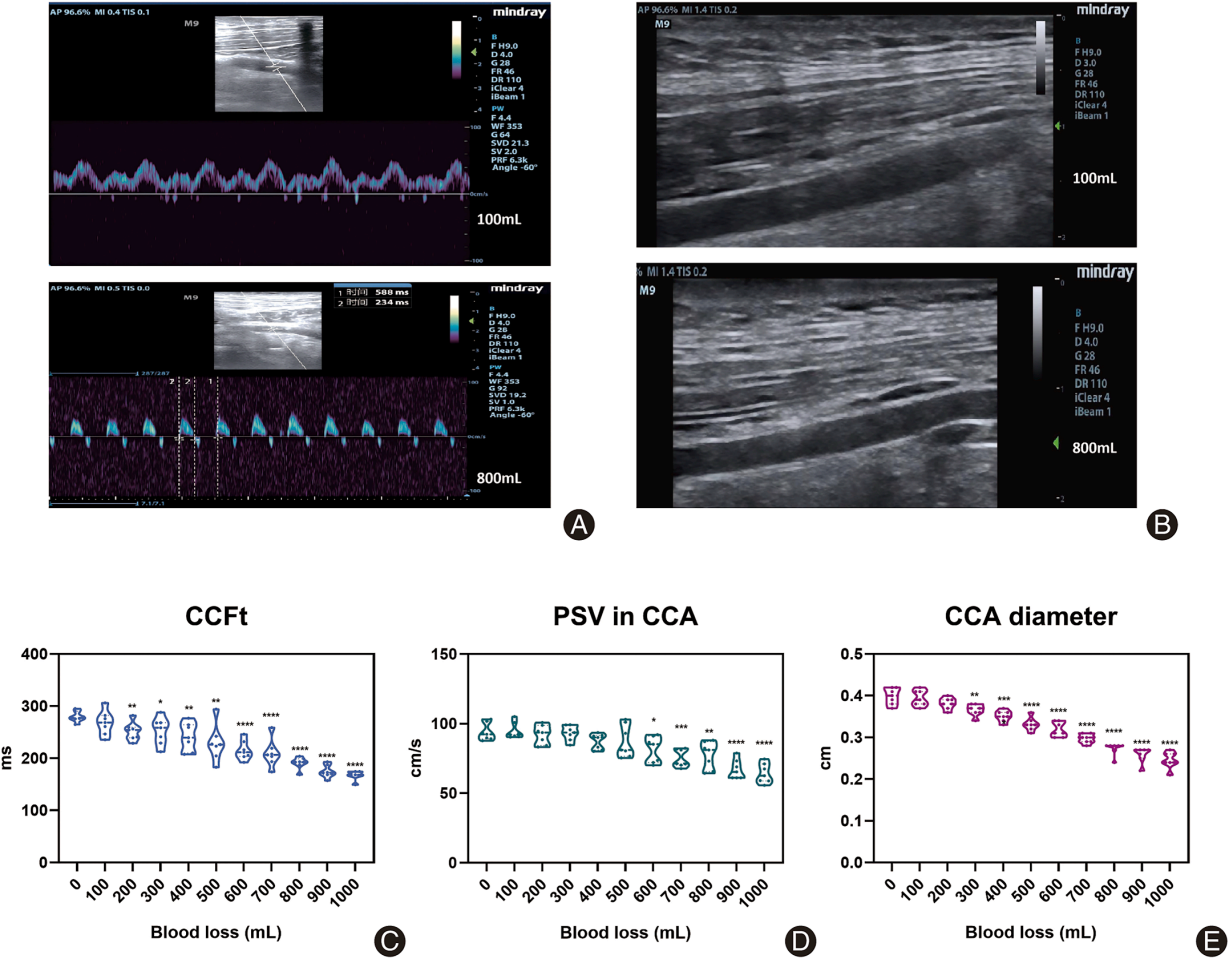
Dynamic evolution of carotid ultrasound imaging characteristics and hemodynamic parameters during graded hemorrhage in experimental animals. (A) Representative changes in carotid pulsed-wave Doppler spectral waveforms. The upper panel displays the waveform at mild blood loss (100 mL), showing normal morphology with preserved diastolic flow; the lower panel demonstrates the waveform at severe blood loss (800 mL), where the systolic peak is significantly narrowed and spiked, and diastolic flow is diminished or absent (cessation of flow). (B) Morphological changes in the long-axis 2-dimensional ultrasound view of the carotid artery. Compared to the filled state at 100 mL blood loss (upper), the carotid lumen exhibits significant collapse at 800 mL blood loss (lower). (C-E) Line graphs illustrating the trends of CCFt, PSV, and CCA diameter relative to cumulative blood loss (0 – 1000 mL). The graphs show a progressive declining trend in CCFt, PSV, and diameter as blood loss increases. Statistical significance versus baseline (0 mL): ∗*p* < 0.05, ∗∗*p* < 0.01, ∗∗∗*p* < 0.001, ∗∗∗∗*p* < 0.0001. CCFt: carotid corrected flow time; PSV: peak systolic velocity; CCA: common carotid artery.

Animals were fasted for 12 h prior to modeling but allowed water ad libitum. On the day of the experiment, anesthesia was induced and maintained via continuous infusion through the marginal ear vein. Animals were secured in a supine position, and a tracheotomy was performed for mechanical ventilation. Under sterile conditions, catheters were placed in the femoral artery (Fig. 1A, ①) for graded blood withdrawal (100 mL per interval) and in the abdominal cavity (Fig. 1A, ②) for the reinjection of autologous blood to simulate hemoperitoneum and hemorrhagic shock (Fig. 1B). Total blood volume was calculated as 65 mL/kg. Referring to the Advanced Trauma Life Support (10th Edition) classification for hemorrhagic shock,^11^ Class I, II, and III hemorrhage models were established, corresponding to blood losses of <15% (<300 mL), 15% – 30% (300 – 600 mL), and >30% (>600 mL), respectively. After cumulative blood loss reached 1000 mL and data acquisition was complete, euthanasia was performed via intravenous injection of an overdose of pentobarbital.

#### 2.1.3 Vital signs monitoring

Systolic blood pressure (SBP), diastolic blood pressure (DBP), and HR were continuously monitored and recorded before injury and after each blood withdrawal. The shock index (SI) was calculated as HR/SBP.

#### 2.1.4 Carotid ultrasound examination

A Mindray M9 portable color Doppler ultrasound system with an L12-4s high-frequency linear array probe (9.0 MHz) was used. Prior to injury and after each withdrawal, the common carotid artery (CCA) was scanned longitudinally from the base of the neck to the angle of the mandible. Pulse wave Doppler sampling was performed at the mid-segment of the CCA, at least 1 cm proximal to the carotid bulb. The angle correction cursor was adjusted to ensure the angle between the ultrasound beam and blood flow was <60°. At least 5 consecutive cardiac cycles with clear waveforms were recorded for each measurement. Bilateral parameters were recorded, including CCA diameter, peak systolic velocity (PSV), systolic time (ST), and cycle time (CT). CCFt was calculated using Bazett's formula: CCFt = ST/ $\sqrt{\text{CT}}$. ST was measured in milliseconds (ms), and CT was measured in seconds (s). The average values of the left and right sides were used for statistical analysis (Fig. 1C).

### 2.2 Clinical trial

#### 2.2.1 Study population

This exploratory case-control study enrolled 18 patients with hemorrhagic shock admitted to our intensive care unit (ICU) between November 2024 and February 2026. This study strictly adhered to the principles of the Declaration of Helsinki and was approved by the Institutional Ethics Committee of Daping Hospital (Approval No. 2025310), and obtained written informed consent from all participants. Case group inclusion criteria: definite diagnosis of hemorrhagic shock due to etiologies including, but not limited to, major hemorrhage, burns, or open fractures. Case group exclusion criteria: (1) inability to complete carotid ultrasound or unsatisfactory image quality; (2) severe arrhythmia; (3) presence of carotid plaque or significant stenosis; (4) incomplete clinical data.

A control group of 36 non-hemorrhagic shock patients was selected during the same period. Efforts were made to exclude major confounders affecting hemodynamics, including severe arrhythmia, significant carotid disease, and unstable BP. However, matching for all comorbidities and medication use (e.g., vasoactive agents) was not fully feasible due to the exploratory design. Control group inclusion criteria: (1) no diagnosis of hemorrhagic shock; (2) normal BP (SBP 90 – 140 mmHg, DBP 60 – 90 mmHg); (3) overall stable clinical condition. Control group exclusion criteria: (1) history of major hemorrhage within the past year; (2) abnormal BP; (3) unsatisfactory ultrasound images; (4) severe arrhythmia; (5) carotid plaque or stenosis; (6) incomplete clinical data.

#### 2.2.2 Clinical data collection

Baseline data, including age, sex, baseline BP, red blood cell count (RBC), and hemoglobin (Hb), were collected. Two complex cases in the case group underwent serial carotid ultrasound monitoring during treatment with concurrent lactate measurements.

#### 2.2.3 Carotid ultrasound examination

The equipment and scanning protocol were identical to those described in the animal experiment (Section 2.1.4). All examinations were performed by a sonographer with over 3 years of experience who was blinded to the patient group allocation and clinical information.

### 2.3 Statistical analysis

Graphing and analysis were performed using GraphPad Prism 8.0, and data verification was conducted using SPSS 26.0. Quantitative data were expressed as mean ± standard deviation.

#### 2.3.1 Comparison in animal experiments

Physiological parameters (HR, SBP, SI) and ultrasound parameters (CCFt, PSV, diameter) at different blood loss nodes (0 mL to 1000 mL) were compared using ANOVA with Tukey's test.

#### 2.3.2 Correlation analysis

Pearson correlation analysis was used to evaluate the relationships between CCFt, PSV, CCA diameter, and blood loss volume, and to calculate the correlation coefficient (*R*).

#### 2.3.3 Diagnostic performance analysis

Receiver operating characteristic (ROC) curves were plotted, and the area under the curve (AUC) was used to evaluate diagnostic efficacy. In animal experiments, the abilities of SBP, SI, and CCFt to identify different degrees of hemorrhage (Class I and Class III) were compared. In clinical validation, the diagnostic efficacy of CCFt in distinguishing patients with hemorrhagic shock from controls was assessed. The optimal cutoff values were determined using the maximum Youden index, based on sensitivity and specificity.

#### 2.3.4 Clinical group comparison

Continuous variables (e.g., CCFt, SBP, RBC, Hb) were compared using the Mann-Whitney *U* test; categorical variables (e.g., sex) were compared using Fisher's exact test.

#### 2.3.5 Analysis of dynamic monitoring cases

For the 2 serially monitored patients, descriptive trend analysis was performed; changes in CCFt relative to disease course were plotted without formal statistical inference.

## 3 Results

### 3.1 Establishment of porcine hemorrhagic shock model and vital sign changes

The graded hemorrhagic shock model was successfully established in all 7 pigs. With incremental blood loss, vital signs showed typical changes associated with shock (Fig. 1D and E**)**.

HR rose progressively with blood loss and was significantly increased *vs*. baseline at 500 mL (*p* = 0.015); at 1000 mL, HR reached (180 ± 10) beats/min (Fig. 1D). SBP remained relatively stable during the early stages but declined significantly from baseline at 300 mL (*p* = 0.026). With cumulative loss reaching 1000 mL and falling below 70 mmHg at 1000 mL, indicating decompensation (Fig. 1E). SI responded rapidly to volume changes, increasing significantly in the early stage (200 mL, *p* = 0.008) and exceeding 2.0 at the endpoint (1000 mL), consistent with severe circulatory failure (Fig. 1F).

### 3.2 Changes in carotid ultrasound parameters and correlation with blood loss

Carotid ultrasound revealed sensitive changes in vascular morphology and hemodynamic parameters corresponding to reduced effective circulating volume. Spectral Doppler showed progressive narrowing and peaking of the systolic waveform, accompanied by attenuation and eventual loss of diastolic flow in severe hemorrhage (Fig. 2A). Two-dimensional imaging revealed marked luminal collapse at 800 mL compared with 100 mL (Fig. 2B).

CCFt demonstrated a pronounced shortening as blood loss increased (Fig. 2C). At the stage of mild hemorrhage (200 mL), CCFt was significantly reduced from baseline (*p* = 0.003). At 1000 mL, CCFt decreased to around 167.4 ms from a preinjury value of 279.9 ms (*p* < 0.001), indicating high sensitivity to blood volume change. PSV and carotid diameter also decreased significantly as blood loss increased. PSV remained relatively stable early on but showed a statistically significant decline at 600 mL blood loss (*p* = 0.013, Fig. 2D). Carotid diameter showed a significant reduction at 300 mL blood loss (*p* = 0.004, Fig. 2E). At 1000 mL, both PSV and diameter were markedly lower than baseline (*p* < 0.001).

**Fig. 3:**
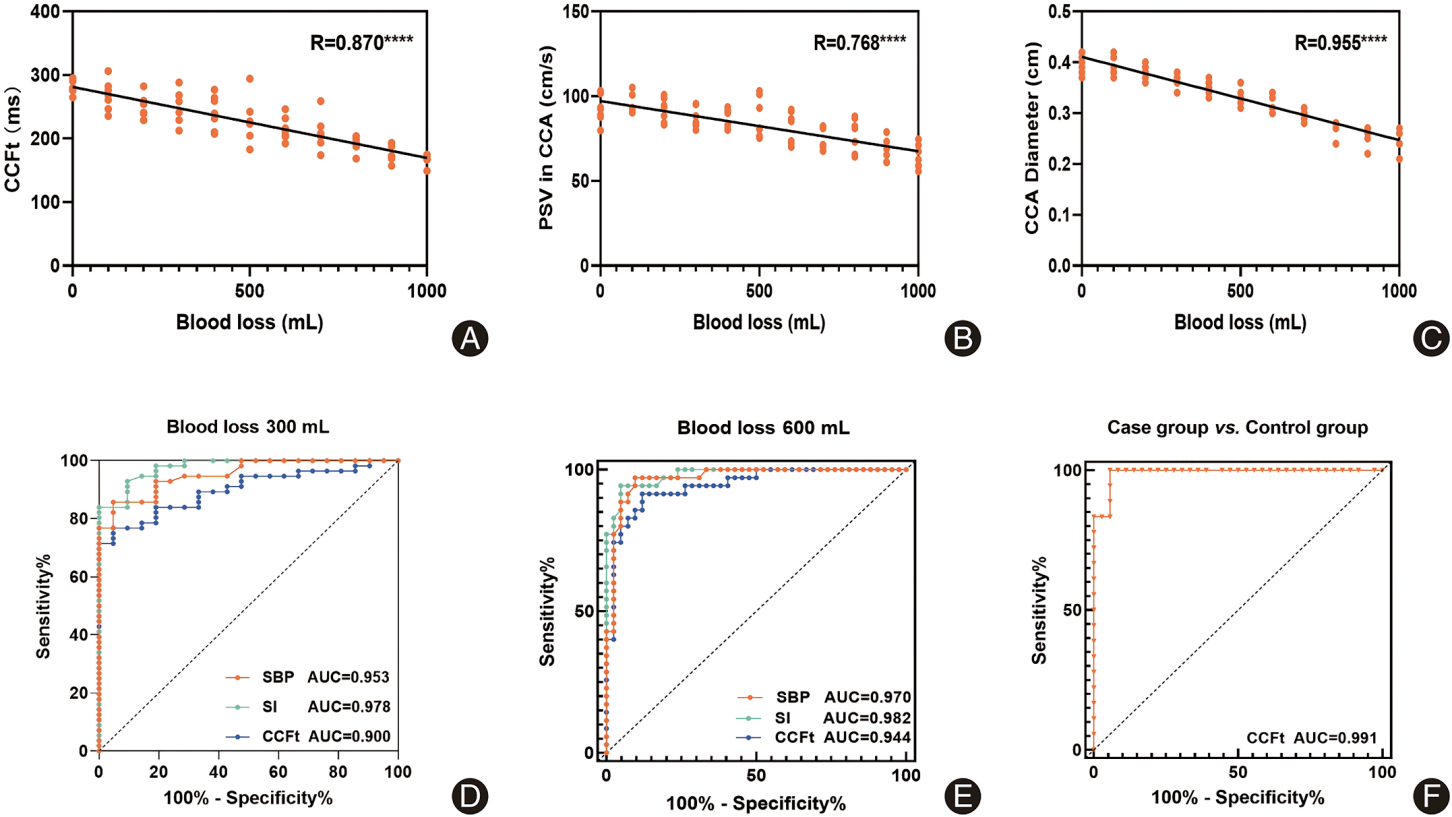
Correlation analysis of carotid ultrasound parameters with blood loss volume and diagnostic efficacy for varying degrees of hemorrhage. (A-C) Pearson correlation scatter plots showing the relationship between cumulative blood loss (0 – 1000 mL) and CCFt, PSV, and CCA diameter. The plots demonstrate that CCFt (*R* = 0.870), PSV (*R* = 0.768), and diameter (*R* = 0.955) are all significantly negatively correlated with blood loss (*p* < 0.001). (D-E) ROC curve analyses comparing the diagnostic value of CCFt, SI, and SBP at different stages of hemorrhage. (D) ROC curves for diagnosing early/mild blood loss (<300 mL); the AUC for CCFt is 0.900. (E) ROC curves for diagnosing severe blood loss (≥600 mL); the AUC for CCFt increases to 0.944, approaching that of SI (0.982) and SBP (0.970). (F) ROC curve for CCFt in distinguishing patients with hemorrhagic shock (*n* = 18) from non-shock controls (*n* = 36) in the clinical study. The analysis indicates high diagnostic accuracy for CCFt, with an AUC of 0.991 (*p* < 0.001). ∗∗∗∗*p* < 0.0001. CCFt: carotid corrected flow time; PSV: peak systolic velocity; CCA: common carotid artery; ROC: receiver operating characteristic; SI: shock index; SBP: systolic blood pressure; AUC: area under the curve.

Pearson correlation analysis revealed significant linear correlations between carotid ultrasound parameters and blood loss volume. Pearson correlation revealed strong negative correlations between carotid parameters and blood loss: CCFt (*R* = 0.870, *p* < 0.001, Fig. 3A), PSV (*R* = 0.768, *p* < 0.001, Fig. 3B), and diameter (*R* = 0.955, *p* < 0.001, Fig. 3C).

### 3.3 Diagnostic efficacy of CCFt for different degrees of hemorrhage

ROC curve analysis demonstrated high diagnostic value of CCFt in assessing hemorrhage severity (Fig. 3D and E). For early/mild blood loss (<300 mL), CCFt yielded an AUC of 0.900. Although slightly lower than SI (AUC = 0.978) and SBP (AUC = 0.953), CCFt displayed excellent early warning capability (Fig. 3D). For severe hemorrhage (≥600 mL, CCFt's AUC increased to 0.944, approaching the performance of SBP (AUC = 0.970) and SI (AUC = 0.982) with no statistically significant differences among these measures (Fig. 3E).

### 3.4 CCFt differences between hemorrhagic shock patients and controls

Eighteen hemorrhagic shock patients and 36 controls were analyzed. Baseline characteristics and values for CCFt, SBP, RBC, and Hb are summarized in Table 1. Age and sex did not differ significantly between groups. RBC, Hb, and SBP were significantly lower in the shock group than in controls (*p* < 0.001). CCFt was also markedly reduced in patients with shock (*p* < 0.001). ROC analysis indicated excellent discriminative performance for CCFt in identifying hemorrhagic shock (AUC = 0.991, *p* < 0.001, Fig. 3F); at a cutoff of 311 ms, sensitivity was 83% and specificity 97%.

**Table 1:** Comparison of clinical parameters and CCFt between the 2 groups.

Clinical parameters	Case group (*n* = 18)	Control group (*n* = 36)	*p* value
Age	52.7 ± 14.3	54.9 ± 11.9	0.551
Male	13 (72.2)	25 (69.4)	0.548
SBP (mmHg)	93.2 ± 13.1	121.2 ± 12.0	<0.001
RBC (×10^12^/L)	3.12 ± 0.70	4.90 ± 0.50	<0.001
Hb (g/L)	92.3 ± 17.4	146.6 ± 15.2	<0.001
CCFt (ms)	283.3 ± 24.6	350.8 ± 19.2	<0.001

Data presented as *n* (%) or mean ± standard deviation.

CCFt: carotid corrected flow time; SBP: systolic blood pressure; RBC: red blood cell count; Hb: hemoglobin.

### 3.5 Dynamic monitoring of clinical cases

**Fig. 4:**
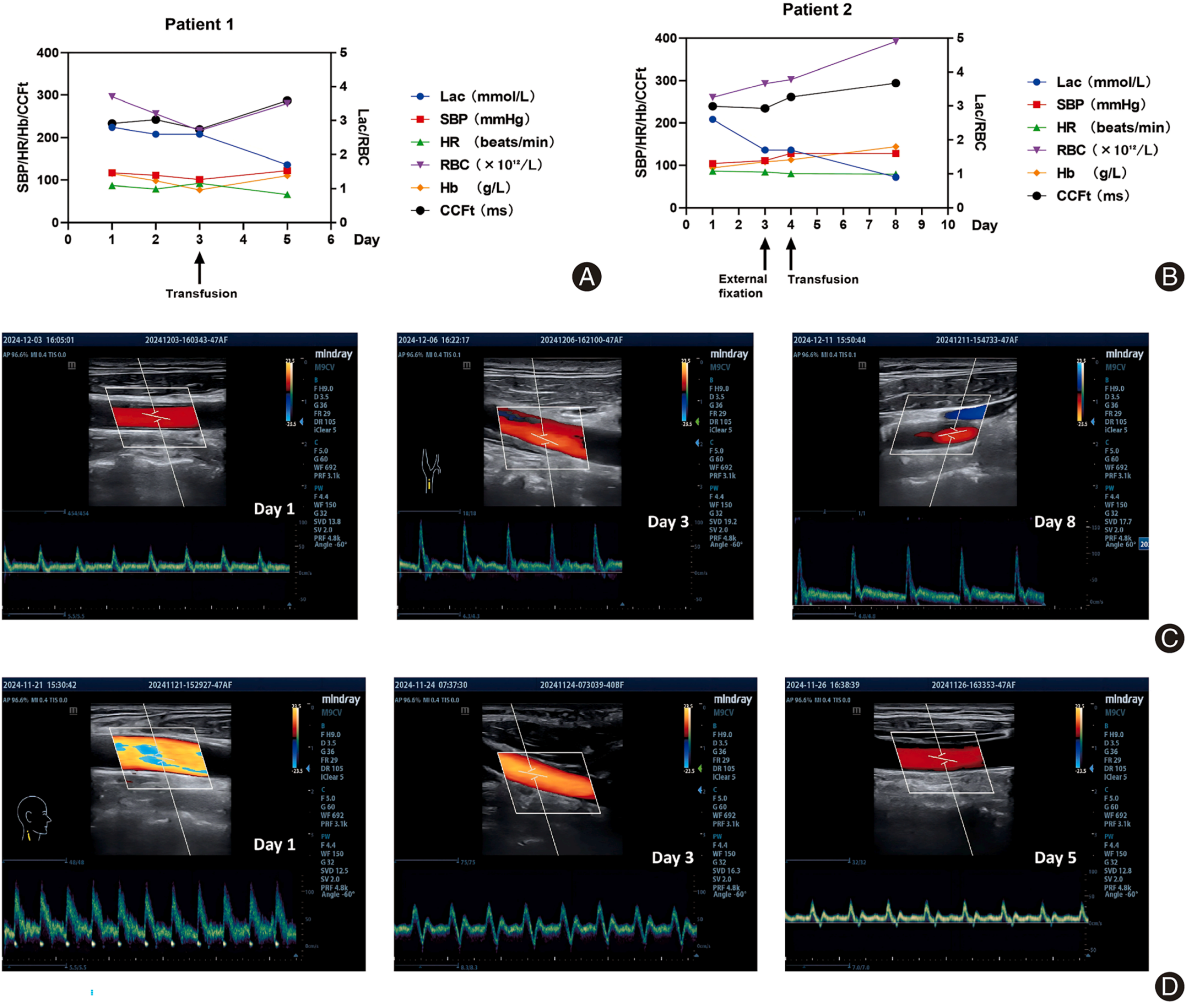
Dynamic monitoring of hemodynamic and metabolic indices and representative ultrasound images in patients with hemorrhagic shock during treatment. (A-B) Multi-parameter dynamic trend charts for 2 representative hemorrhagic shock patients during resuscitation, with key clinical interventions (e.g., transfusion, surgery) annotated along the timeline. The graphs display the time-course changes in SBP, HR, Hb, RBC, Lac, and CCFt. (A) Patient 1 (upper gastrointestinal bleeding): On Days 1, 3, and 5 of admission, following hemostasis and transfusion, Hb and RBC recovered, lactate cleared, and CCFt significantly prolonged (recovering from a nadir of 219.0 ms to 286.9 ms). (B) Patient 2 (open fracture): From Day 1 to Day 8 of admission, following surgery and transfusion support, CCFt showed an improving trend consistent with the rise in Hb and the decline in Lac, eventually restoring to 293.6 ms. (C-D) Comparison of carotid artery pulsed-wave Doppler spectral morphology between Patient 1 (C) and Patient 2 (D) at different treatment stages. During the shock stage, the spectrum presents a narrow and sharp systolic peak with a steep ascending limb and a rapidly descending limb, corresponding to a significant shortening of the CCFt. With the recovery of effective circulating blood volume, the spectral waveform broadens markedly, and CCFt prolongs to the normal range. SBP: systolic blood pressure; HR: heart rate; Hb: Hemoglobin; RBC: red blood cell count; CCFt: carotid corrected flow time; SI: shock index.

Two ICU patients underwent serial monitoring to evaluate CCFt dynamics during treatment. Case 1 (middle-aged male, gastrointestinal bleeding, Fig. 4A): On Day 1, RBC and Hb were significantly reduced, lactate was elevated, and CCFt was reduced (233.0 ms). By Day 3, with ongoing bleeding, Hb dropped to 87 g/L, and CCFt shortened to 219.0 ms; hemostasis and transfusion were initiated. By Day 5, perfusion improved, Hb recovered (110 g/L), lactate normalized, and CCFt increased to 286.9 ms. Case 2 (young female, open fracture, Fig. 4B): on Day 1, Hb was 87 g/L, and CCFt was 239.0 ms. By Day 3, after external fixation, Hb rose slightly (108 g/L) and lactate decreased with minimal CCFt change (234.0 ms). Following transfusion on Day 4, Hb reached 113 g/L, and CCFt increased to 260.5 ms. By Day 8, circulation was restored (Hb 144 g/L), lactate normalized, and CCFt increased to 293.6 ms. Notably, BP and HR remained within normal ranges throughout treatment in both cases, likely due to active life support. Overall, across both cases, CCFt demonstrated a consistent temporal relationship with major therapeutic interventions, particularly transfusion and hemostatic management, highlighting its potential as a real-time indicator of volume status. Carotid Doppler waveforms (Fig. 4C and D) showed narrow, spiked systolic upstrokes with rapid decline during shock, corresponding to shortened CCFt; with resuscitation, the spectral waveform broadened and CCFt prolonged.

## 4 Discussion

Hemorrhagic shock is a primary cause of early mortality in trauma patients, making hemodynamic monitoring a central component of clinical decision-making.^2^^,^^12^ Existing monitoring modalities have important limitations. Static indicators such as BP and HR may be preserved by neurohumoral compensation during early shock, exhibiting significant diagnostic lag and often failing to detect abnormalities until blood loss exceeds around 30%.^13^ Central venous pressure has been shown to be a poor predictor of fluid responsiveness in meta-analyses.^14^ Biochemical markers, such as lactate, reflect downstream metabolic consequences of hypoxia and lack real-time responsiveness. Invasive monitoring (e.g., pulmonary artery catheter and pulse indicator continuous cardiac output) is accurate but invasive, complex, and carries risks of complications; it is unsuitable for pre-hospital settings.^15^ Therefore, non-invasive visual assessment tools that provide timely information are urgently needed.

According to the Frank-Starling mechanism, reduced preload in hypovolemia leads to decreased ventricular filling and stroke volume, mechanically shortening ejection time. The carotid artery, being proximal to the aortic arch, represents a reliable window to central hemodynamics;^8^ hence, CCFt reflects both cardiac filling and ventricular-arterial coupling. Prior studies using blood donation models^16^ and lower-body negative-pressure models^17^ have confirmed that CCFt shortens with reduced preload and prolongs with resuscitation. Our porcine model confirms a near-linear decline of CCFt with incremental blood loss from mild (300 mL) to severe (1000 mL) hemorrhage. Interestingly, carotid diameter showed the strongest correlation with blood loss (*R* = 0.955), in contrast to some human studies that reported minimal changes in diameter with BP variation.^18^ This discrepancy may be due to the higher vascular compliance in experimental pigs or the fact that our study involved severe blood loss (>40% of total volume), far exceeding routine human simulation experiments. These findings suggest that vascular collapse, in addition to reduced flow velocity, is a salient morphological change in severe hemorrhage and warrants further exploration.

Early identification of compensated shock remains a challenge in trauma care. Our animal data indicate that CCFt detects significant changes at early blood loss (<300 mL), with diagnostic performance (AUC = 0.900) comparable to conventional indices and superior to HR alone. This aligns with findings by Barjaktarevic et al.^19^ in undifferentiated shock, supporting CCFt as a sensitive surrogate for stroke volume. Clinically, we identified 311 ms as an optimal cutoff for diagnosing hemorrhagic shock; this threshold is lower than values reported for predicting post-induction hypotension in elective surgical cohorts (e.g., 334 ms, 379 ms),^20^^,^^21^ likely reflecting that our cohort consisted of patients with confirmed hemorrhagic shock pathology, representing a more severe physiological derangement than preoperative patients in relatively healthy states. Our clinical AUC (0.991) validates CCFt's discriminative capacity and, through serial case observations, demonstrates that CCFt trends closely parallel Hb recovery and lactate clearance during resuscitation. Notably, CCFt fluctuated concordantly with metabolic recovery even when BP and HR remained within normal ranges, supporting its potential utility to detect “occult shock” and guide precision resuscitation—particularly when conventional vital signs may be deceptively normal due to compensatory mechanisms or vasoactive agents. This concurs with Kenny's work supporting ultrasound-based monitoring of resuscitation responses.^22^

Limitations: First, the sample size was limited due to ethical constraints in the animal model and the exploratory nature of this single-center clinical study. Although the observed effect sizes were robust, the relatively small cohort may limit statistical power and generalizability. Therefore, larger, multicenter studies with formal sample size estimation are required to validate these findings. Second, operator dependence exists; although performed by senior physicians, ultrasound measurements are inherently subjective, particularly regarding Doppler angle correction. Future research could evaluate AI-assisted measurement to mitigate subjectivity. Third, although major confounders such as severe arrhythmia and carotid stenosis were excluded, the effects of medications (e.g., beta-blockers or vasoactive agents), which may influence cardiac cycle dynamics and CCFt, were not systematically controlled and may represent a potential confounder.

In conclusion, using a graded hemorrhage model in Bama miniature pigs and a clinical case–control validation, we systematically demonstrate that CCFt correlates strongly with blood loss and exhibits characteristic shortening during the compensated phase prior to hypotension. CCFt also dynamically reflects hemodynamic recovery during treatment. These findings support CCFt as a promising non-invasive adjunct for bedside assessment of volume status and for guiding fluid resuscitation in emergency and ICU settings.

## CRediT authorship contribution statement

**Huan Yang:** Writing – original draft, Formal analysis, Data curation. **Hao Tang:** Validation, Data curation. **Yao Xiao:** Validation, Software, Methodology. **Yingying Zhang:** Software, Investigation, Data curation. **Xiao Chen:** Validation, Resources. **Chang Liu:** Validation, Software. **Yi Zhang:** Validation, Methodology. **Yusheng Zhang:** Software, Investigation. **Jingqin Fang:** Supervision, Project administration. **Lianyang Zhang:** Project administration, Funding acquisition. **Tao Li:** Supervision, Conceptualization. **Dong Han:** Writing – review & editing, Methodology, Data curation, Conceptualization.

## Ethical statement

The animal research protocol was approved by the Animal Welfare and Ethical Review Committee of the Army Medical University (Approval No. 20257051). All animal experiments were conducted in accordance with the National Institutes of Health Guide for the Care and Use of Laboratory Animals, and the study adhered to the ARRIVE guidelines. The clinical study was approved by the Institutional Ethics Committee of Daping Hospital (Approval No. 2025310), and written informed consent was obtained from all participants.

## Funding

This work was supported by the 10.13039/501100012166National Key Research and Development Program of China (Research on Intelligent Injury Sensing and Early Warning Technology and Equipment, Grant No. 2023YFC3011800).

## Declaration of competing interest

All authors state that there is no conflict of interest.
